# Comparison of the Effect of Two Hyaluronic Acid Preparations on Fibroblast and Endothelial Cell Functions Related to Angiogenesis

**DOI:** 10.3390/cells8121479

**Published:** 2019-11-21

**Authors:** Valerio Ciccone, Marco Zazzetta, Lucia Morbidelli

**Affiliations:** 1 Department of Life Sciences, University of Siena, 53100 Siena, Italy; ciccone3@student.unisi.it; 2 Regenyal Laboratories Srl, 63074 San Benedetto del Tronto (AP), Italy; farmacovigilanza@regenyal.eu

**Keywords:** hyaluronic acid, angiogenesis, fibroblasts

## Abstract

Hyaluronic acid (HA) is used in substitutive and aesthetic medicine with various applications. Ultrapure absorbable HA (Bioregen^®^) and a mix of reticulated and free low molecular weight HA (Regenyal Idea Bioexpander^®^) (both provided by Regenyal Laboratories Srl, San Benedetto del Tronto (AP), Italy) represent a reliable hydrating device and skin filler, useful for skin blemishes, lines and wrinkles, and lip widening, respectively. The commercial products are known for their safety, but data on the molecular, cellular, and tissue responses are lacking. We aimed to evaluate the bioavailability and the pro-angiogenic features of the products Bioregen^®^ and Bioexpander^®^ in vitro on cultured endothelial cells (ECs) and dermal fibroblasts in vivo when injected into experimental animals. When added to fibroblasts and ECs, Bioexpander^®^ induced cell migration. The two HA preparations were well tolerated, while a transient proangiogenic behavior of Bioexpander^®^, when implanted subcutaneously in mice, was found. The neovascular response was evident in the first week with higher levels of VEGF and FGF-2 before undergoing regression. In conclusion, our data strengthen the safety of HA synthetic preparations both in vitro and in vivo. Even if a proangiogenic response is documented, it is modest and transient, leading to tissue recovery and absence of an inflammatory infiltrate.

## 1. Introduction

Hyaluronic acid (HA) (a nonsulfated glycosaminoglycan consisting of repeated disaccharide units of D-glucoronic acid and N-acetylglucosamine), glycans (nHA), and oligosaccharides (oHA) exert different effects on the biological functions of cells and tissues, such as in the connective, epithelial, and neural tissues [1,2]. HA plays an important role in maintaining the extracellular matrix structure and regulating intercellular activities, such as cell–cell attachment and cell adhesion, by engaging membrane-bound ligands, glycans, and other HA-binding proteins. Moreover, HA occupies a large hydrodynamic volume that greatly influences the hydration and physical properties of tissues [3].

HA is a naturally occurring glycosaminoglycan which, by virtue of its viscosity, elasticity, and other rheological properties (particularly for its biocompatibility), acts as a lubricating and shock absorbing fluid in skin and joints and as an ocular lubricant. HA is well tolerated and nonimmunogenic and for these reasons its potential use in medicine is wide-ranging. In fact, the medical use of HA covers clinical use in ophthalmology, such as for cataract surgery, retinal reattachment, corneal transplantation, trabeculectomy in glaucoma, joint diseases (particularly osteoarthritis and rheumatoid arthritis), and adjuvant in wound healing [4].

In addition to its use in conventional medicine, in recent years HA has been commonly used in aesthetic medicine as filler and in the treatment of the signs of aging and in resolving skin blemishes [5,6]. Some features of facial aging are the development of facial lines and reduction of lip volume. Fillers include autologous fat, bovine serum collagen, autologous and allogeneic human collagen, and expanded polytetrafluoroethylene. HA fillers have been proposed as alternatives to other temporary skin fillers for treating facial skin lines and for providing lip augmentation. The advantages of HA in aesthetic medicine are that it is easy to produce, modifiable, hydrophilic, and nonadhesive, and naturally biodegradable. Injectable soft tissue substitutes provide an affordable, nonsurgical alternative for correcting contour defects in facial skin. Hyaluronan has all the characteristics for achieving this goal; it is biocompatible, nonpyrogenic, nontoxic, and stable after injection [5,6].

It is noteworthy to observe that HA activities strongly depend on polymer size and the degree of cross-linking. High molecular weight HA (HMWHA) shows anti-inflammatory and immune suppressive activities and induces tissue reparative processes as described in wound healing [7]. It has been demonstrated that high molecular weight HA (i.e., 1000–10,000 kDa) inhibits angiogenesis and maintains stable vessels [2,8]. In an opposing fashion, the fragments of HA, oligosaccharides with a size of less than 200 kDa, show the capacity to induce the inflammatory process as well as angiogenesis through interaction with specific receptors, such as CD44 and the receptor for hyaluronan-mediated motility (RHAMM). This is also accomplished by recruiting stromal cells and through stimulating the proliferation and migration of ECs [9,10].

At tissue level, vascularization and organization of the stromal components are perquisites for tissue homeostasis and the biocompatibility of synthetic medical devices. An adequate blood supply guarantees cell survival and correct biochemical functioning without oedema formation, inflammatory cell infiltration, and scar development [11,12]. ECs are very sensitive to exogenous biomaterials and mechano–physical stimuli in general [13,14,15]. On the other hand, stromal cells like fibroblasts, the most common cells of connective tissue, represent more robust cells. Fibroblasts are the main producers of collagen and other extracellular matrix proteins organized in the structural framework of animal tissues. Their proliferation and synthetic activity should, however, be finely tuned to avoid a lack of biocompatibility, fibrous scar formation, or angiogenic factor dysregulation [16]. Indeed, a persistent and aberrant angiogenesis is a negative feature for tissue wellness and can be responsible for severe fibrosis and scarring, besides neoplastic disease development [11,17].

The aim of the study was to evaluate the bioavailability and the pro-angiogenic features of two commercial products, Bioregen^®^ (resorbable HA) and Regenyal Idea Bioexpander^®^ (a mix of reticulated and free low molecular weight HA) in vitro on both cultured endothelial cells (ECs) and dermal fibroblasts and in vivo when implanted into experimental animals. The expression of inflammatory markers and the unbalance of proangiogenic factors (such as vascular endothelial growth factor-VEGF and fibroblast growth factor-2, FGF-2) vs. antiangiogenic proteins (i.e., endostatin) was monitored during this study.

## 2. Materials and Methods

### 2.1. Cell Cultures

Human umbilical vein endothelial cells (HUVEC) were purchased form Lonza (Basel, Switzerland) and grown in complete endothelial growth medium (EGM-2) (Lonza, Basel, Switzerland). Some experiments were performed using human dermal blood endothelial cells (HDBEC) purchased from Promocell (Heidelberg, Germany) and grown in complete endothelial cell basal medium (MV2; Promocell, Heidelberg, Germany). Both cell lines were supplemented with 10% fetal bovine serum (FBS) (Hyclone, Euroclone, Milan, Italy).

Normal human dermal fibroblasts (NHDFs) were from Lonza (Verviers, Belgium). The medium used was fibroblast basal medium (FBM) supplemented with 10% FBS. Cells were cultured at 37 °C in 5% CO_2_. Both endothelial cells and fibroblasts were split 1:3 twice a week and used until passage 10.

### 2.2. Materials

In this study, we assessed the activity of Bioregen^®^—an ultrapure multifractional adsorbable HA—and Regenyal Idea Bioexpander^®^—a mix of reticulated and intercalated free low molecular weight HA—both provided by Regenyal Laboratories Srl, S. Benedetto del Tronto (AP), Italy. Specifically, Bioregen^®^ is a mix of five different but not crosslinked molecular weights of HA sodium salt of non-animal origin (1 MDa, 500 kDa, 200 kDa, 100 kDa, and 2 kDa) obtained by bacterial fermentation. Fragmentation was carried out by implementing heat and ultrasounds in a highly alkaline environment, starting from the fraction of 1 MDa. Analytical characterization was performed as described in [18]. The HA concentration in Bioregen^®^ is 16 mg/mL. Bioexpander^®^ is a mix of three different molecular weights of HA: 2 and 1 MDa crosslinked together with butanediol diglycidyl ether, while 500 kDa HA is not crosslinked but intercalated (patented technology). The final HA concentration in Bioexpander^®^ is 25 mg/mL.

Unless indicated, the ready to use commercially available preparations were used, otherwise each original preparation was weighted in an analytical balance to prepare the appropriate concentrations in cell culture medium and reported in the results as [mg/mL].

### 2.3. Cell Survival and Proliferation

Fibroblast and EC proliferation was evaluated according to the same protocol. One thousand cells/well (of a 96-well multiplate) were left to adhere in 10% serum for 3–4 h and then HA preparations (used at increasing concentrations in the range 0.1–10 mg/mL) were added in a medium with 2% serum which represented the basal control condition. All experimental points using cells from the single culture plate were run in triplicate. After 2 days, cells were fixed, stained with H&E and randomly counted at 20× original magnification in 5 fields [19,20]. Data are reported as number of cells counted/well.

### 2.4. In Vitro Cell Migration by Scratch Assay

Cells (monocultures of fibroblasts or ECs) were seeded into 24-well plates (1 × 10^5^ cells/well) and, after confluence, were scratched using a sterile tip. Once gently washed to remove debris cells were treated with HA preparations diluted in a medium with 2% serum for 18 h in the presence of the antimitotic ARA-C (25 ug/mL) (Sigma-Aldrich, St. Louis, MO). This was to evaluate only cell migration. The results are expressed as the percentage of wound area with respect to time to 0, measured by image analysis software [20].

### 2.5. Subcutaneous Plug Implant and Analysis

The in vivo angiogenesis assay was performed as previously described [20]. Eight C57 black mice (20–25 g) were kept in temperature- and humidity-controlled rooms (22 °C, 50%) with lights on from 07:00 to 19:00 h, and water and food available ad libitum. The investigation was conducted in accordance with the ethical standards and according to the Declaration of Helsinki and the Italian law (Legislative Decree no.26, 4 March 2014), which acknowledges the European Directive 2010/63/UE, being approved by the authors’ institutional review board and the Italian Ministry of Health (authorization n. 55/2017-PR).

The mice were SC injected in the dorsal midline region with 0.4 mL of Bioregen^®^ or Regenyal Idea Bioexpander^®^. In some of the experiments, the HA preparations were mixed with Matrigel (Becton Dickinson, Waltham, MA, USA) (1:1, v:v) on ice. At different times, compatible with the biomaterial half-life once injected (1–9 days for the absorbable Bioregen^®^ and 1–4 weeks for Bioexander^®^), mice were sacrificed by CO_2_ inhalation and implants were harvested. The implants were re-suspended in 1 mL of Drabkin’s reagent (Sigma-Aldrich, St. Louis, MO) for 18 h on ice, and haemoglobin concentration was determined by absorbance at 540 nm and compared with a standard curve (Sigma-Aldrich, St. Louis, MO). Some plugs were stored at −80 °C for protein extraction and Western blot analysis.

### 2.6. In Vivo Rabbit Corneal Pocket Assay

Angiogenesis was studied in the cornea of male New Zealand white rabbits (Charles River, Italy) as described in [21]. The investigation was conducted in accordance with the ethical standards and according to the Declaration of Helsinki and the Italian law (Legislative Decree no.26, 4 March 2014), being approved by the authors’ institutional review board and the Italian Ministry of Health (authorization n. 148/2015-PR). Rabbits (1.5–2.0 Kg, 2 animals) were anaesthetized with xilazine solution (20 mg/mL, Xilor^®^, BIO 98 Srl, Milan, Italy) used at the dose of 0.5 mL (i.m.) and followed by tiletamine and zolazepam (10 mg/mL; Zoletil^®^ Virbac Srl, Milan, Italy) given at the dose of 5 mg/kg (i.m.). The deepness of anaesthesia was checked as reflex to pressure. Each eye was protruded by the use of a dental dam and a local anaesthetic (0.4% benoxinate) was instilled just before surgery. The Regenyal Idea Bioexpander^®^ implantation procedure started with a linear intrastromal incision, parallel to the corneoscleral limbus (linear keratotomy), using a surgical blade. The corneal pocket for the plug implant was produced with a 1.5 mm pliable silver spatula with a smooth edge blade in the lower half of the cornea. The HA gel was introduced with a syringe through the keratotomy line, parallel to and under the corneal epithelium in the external third of the stroma, up to 2 mm from the limbus [21,22]. The corneas were observed every two other days for 2 weeks after implant on unanesthetized living animals, and digital images were taken by means of a slit-lamp stereomicroscope.

### 2.7. Western Blot for Protein Expression

Western blot analysis was performed on the depolymerized plugs of HA or in cell culture lysates. The plugs were isolated from the mice and depolymerized. The HA depolymerization was carried out using hyaluronidase (60 U/mL) for 18 h at 37 °C (Sigma Aldrich, St. Louis, MO, USA) in phosphate buffer saline (pH 7.4) [23].

Subconfluent fibroblasts and ECs were seeded in 6 cm diameter petri dishes. After adherence, cells were treated for 18 h with Bioregen^®^ or Regenyal Idea Bioexpander^®^ (at 1 mg/mL concentration) in a medium with 2% serum.

Protein extraction and Western blot were performed as previously described [11,12,13]. Electrophoresis (50 µg of proteins/sample) was carried out in 4–12% Bis-Tris Gels (Life Technologies, Carlsbad, CA, USA). Proteins were then blotted onto nitrocellulose membranes, incubated overnight with antibodies anti FGF-2 (at 1:1000, no. 3196 Cell Signaling Technology, Danvers, MA, USA), anti VEGF (at 1:1000 no. 101-M60 ReliaTech GmbH, Germany), anti-fibronectin (at 1:1000, no. F3648 Sigma Aldrich, St. Louis, MO, USA), anti-CD40 (at 1:500, no. sc-975, Santa Cruz Biotechnology, Dallas, TX, USA), anti-endostatin (at 1:1000 no. 05-579 Sigma Aldrich, St. Louis, MO, USA) and anti-ZO-1 (at 1:1000 no. 610966 BD Biosciences San Jose, CA, USA) and then detected by enhanced chemiluminescence system (Bio-Rad, Hercules, CA, USA). The results were normalized to those obtained using an antibody against β-actin (at 1:10000 no. A5441; Sigma Aldrich, St. Louis, MO, USA).

### 2.8. Data Analysis and Statistical Procedures

The results are either representative or the average of at least three independent experiments done in triplicate starting from different single culture plates. Statistical analysis was performed using ANOVA test followed by Bonferroni test, and Student t test when appropriate (GraphPad). P < 0.05 was considered statistically significant.

## 3. Results

### 3.1. HA Preparations Differentially Promote Fibroblast and Endothelial Cell Proliferation and Migration

**Figure 1:**
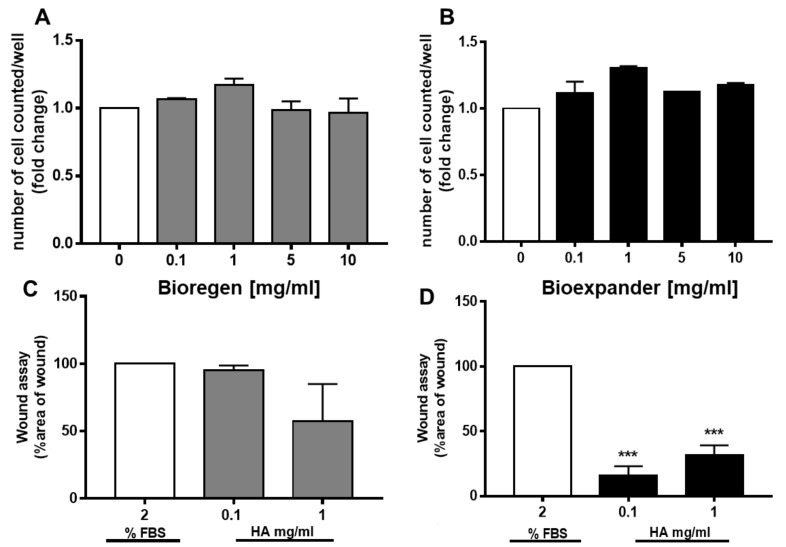
Fibroblast proliferation and migration in response to hyaluronic acid (HA) preparations. (**A**,**B**) Fibroblasts were exposed to increasing concentrations of the HA preparations Bioregen^®^ (grey columns) (**A**) and Bioexpander^®^ (black columns) (**B**) (0.1–10 mg/mL) for 48 h in a medium containing 2% serum. Cell number, index of survival, and proliferation were counted in a blind manner. (n = 3). (**C**,**D**) Migration of adherent cells was evaluated by the scratch assay in confluent fibroblast monolayers. The biomaterials Bioregen^®^ (grey columns) (C) and Bioexpander^®^ (black columns) (**D**) were tested at 0.1 and 1 mg/mL concentrations in the presence of 2% fetal bovine serum (FBS). Graphs report the percentage of wound area with respect to time 0 (n = 3). *** *p* < 0.001 vs. basal condition.

When fibroblasts were exposed to increasing concentrations of both Bioregen^®^ and Bioexpander^®^ for 48 h, no impairment of cell survival was found. The number of cells in the presence of the biomaterial was maintained as in the control condition (medium supplemented with 2% FBS), with a modest increase (in the range of 20–30% cell number) at 1 mg/mL of hyaluronic acid for both products (Figure 1A,B).

The scratch assay was performed on confluent fibroblast cultures in order to evaluate cell motility in the presence of the biomaterial to mimic wound healing. Comparing the two forms of HA, only Bioexpander^®^ was able to induce fibroblast migration, significantly reducing the wound area, while a trend (not significant) toward migration was assessed with Bioregen^®^ (Figure 1C,D).

**Figure 2:**
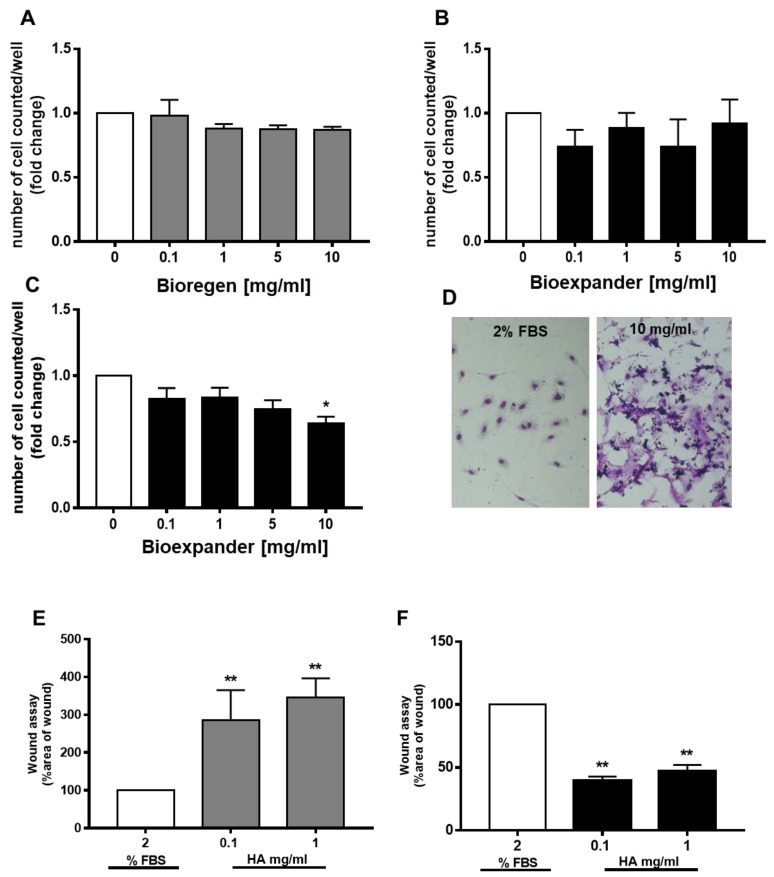
Endothelial cell proliferation and migration are differently affected by HA preparations. (**A**–**C**) Endothelial cells (A and B, human umbilical vein endothelial cells (HUVEC) and C, HDBEC) were exposed to increasing concentrations of Bioregen^®^ (grey columns) and Bioexpander^®^ (black columns) (0.1–10 mg/mL) dissolved in a medium with 2% serum. Cell number, index of survival, and proliferation were counted after 48 h. (n = 3). (**D**) Representative pictures of stained HDBEC in the control condition and following incubation with 10 mg/mL of Bioregen^®^ (4× original quantification). (**E**,**F**) The migration of adherent HUVEC was evaluated by the scratch assay in confluent monolayers. The biomaterials Bioregen^®^ (grey columns) and Bioexpander^®^ (black columns) were tested at 0.1 and 1 mg/mL concentrations in the presence of 2% FBS. Graphs report the percentage of wound area with respect to time 0 (n = 3). * *p* < 0.05 and ** *p* < 0.01 vs. basal condition.

The endothelial cells did not increase their proliferative potential. Sporadic variation in survival was measured on both HUVEC and HDBEC (Figure 2A–C), with a clear and reproducible inhibitory effect by Bioexpander^®^ at 10 mg/mL (Figure 2C). The modulation of survival by Bioexpander^®^ (at the highest concentrations) was accompanied by the physical deposition of the biomaterial on the endothelial cell surface, as evidenced by purple-blue stained material on the cell cultures after cell fixation and H&E staining (Figure 2D).

In the wound healing assay, while a clear increase in cell migration was measured in endothelial cells (HUVEC) exposed to 0.1 and 1 mg/mL of Bioexpander^®^ (Figure 2F), Bioregen^®^ did not induce any migration (Figure 2E). On the contrary, a retard in ECs migration with Bioregen^®^ was clearly monitored (Figure 2E).

In the complex, the results evidence the promigratory effect of Bioexpander^®^ on both fibroblasts and endothelial cells. These results are substantiated by the fact that the scratch assay is performed in the presence of an antimitotic agent, in order to avoid cell proliferation that can mask cell spreading.

### 3.2. HA Preparations Affect the Expression of Angiogenesis Markers In Vitro

**Figure 3:**
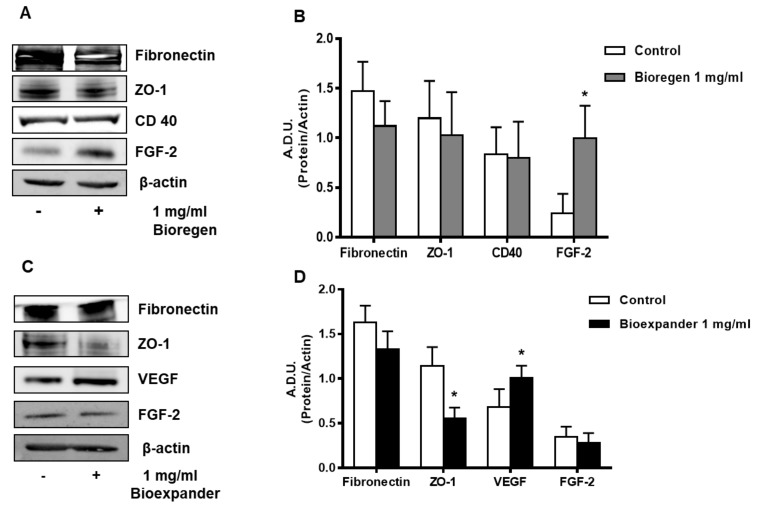
Analysis of the angiogenesis and inflammatory markers following HA exposure of skin fibroblasts. NHDFs were treated for 18 h with 1 mg/mL Bioregen^®^ (grey columns) (**A**,**B**) and Bioexpander^®^ (black columns) (**C**,**D**). In panels (**A**,**C**), a representative blot out of 3 is shown, while panels (**B**,**D**) report protein quantification. Data are reported as A.D.U. of the protein of interest with respect to beta-actin. * *p* < 0.05 vs. basal control condition.

Human skin fibroblasts and ECs were exposed to 1 mg/mL of HA preparations for 18 h and various relevant markers of inflammation and angiogenesis were measured by Western blot. Exposure of fibroblasts to Bioregen^®^ induced an upregulation of FGF-2 and downregulation of the adhesion molecule fibronectin, consistent with a trend to migration (Figure 3A,B). Interestingly, an increase in VEGF, but not FGF-2, expression was found in NHDFs treated with Bioexpander^®^ (Figure 3C,D). Of note, the expression of the cell–cell contact zonula occludens-1 (ZO-1) was reduced by Bioexpander^®^ incubation, thus strengthening the promigratory effect of this HA formulation (Figure 1D).

**Figure 4:**
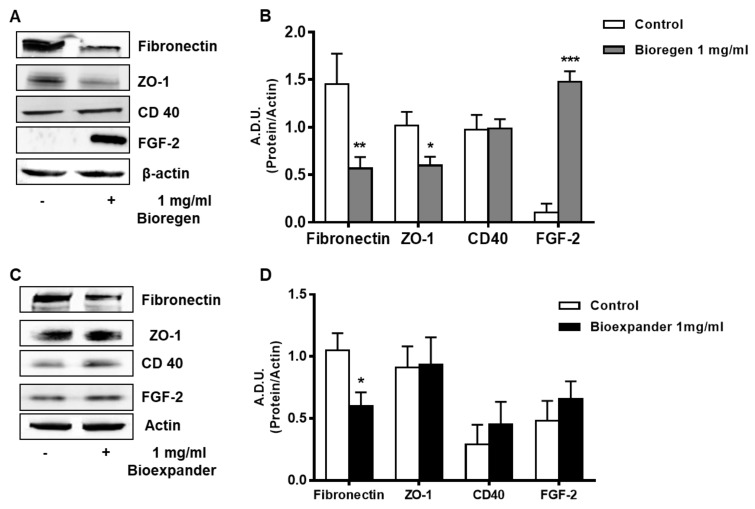
Analysis of the proteins involved in angiogenesis and inflammation in ECs. HUVEC cells were treated for 18 h with the 1 mg/mL HA preparations Bioregen^®^ (**A**,**B**) (grey columns) and Bioexpander^®^ (**C**,**D**) (black columns). In panels (**A**,**C**), a representative blot out of 3 is shown, while panels (**B**,**D**) report protein quantification. Data are reported as A.D.U of the protein of interest with respect to beta-actin. * *p* < 0.05, and ** *p* < 0.01 vs. basal control condition.

In HUVEC exposed to Bioregen^®^, we observed a significant increase in FGF-2 and a reduction of fibronectin and ZO-1 (Figure 4A,B), while Bioexpander^®^ exerted milder effects (Figure 4C,D).

CD-40, a marker of EC activation toward an inflammatory and angiogenic phenotype [12,24,25], was unchanged after exposure of the HUVECs to Bioregen^®^, while it mildly increased following incubation with Bioexpander^®^ (Figure 4).

All these data in their complexes indicate that HA preparations affect the functional and phenotypic responses of endothelial and stromal cells in relation to angiogenesis, but reinforce their biocompatibility since there is a lack of inflammatory signal upregulation.

### 3.3. Induction of In Vivo Angiogenesis

**Figure 5:**
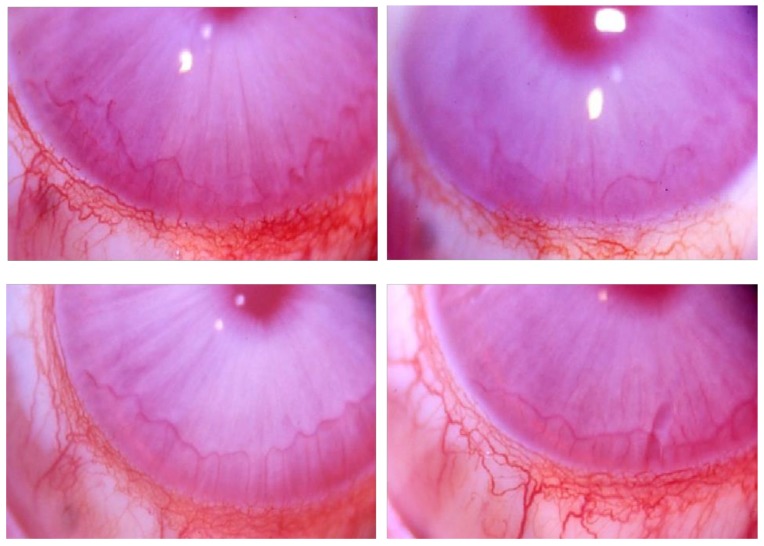
Images of implanted rabbit corneas. The ready to use solution of Bioexpander^®^ was surgically injected in the cornea stroma of albino NZW rabbits under anaesthesia. In the following 2 weeks, corneas remained transparent and no angiogenesis occurred (n = 4). The pictures were taken at day 10. The white spots are reflex artefacts.

Afterwards, HA preparations were tested for direct proangiogenic properties in the avascular rabbit cornea assay and as subcutaneous implants. The biomaterial Bioexpander^®^ implanted inside a micropocket in the rabbit cornea stroma (n = 4 implants) was well tolerated and no adverse effect was observed after ten days (Figure 5). Corneas remained transparent and no angiogenesis occurred, documenting that biomaterial per se is not an angiogenic inducer in an environment where antiangiogenic molecules are predominant and vessels are absent [26].

The process of angiogenesis was further evaluated following the subcutaneous implant in mice through morphological and biochemical analysis of the harvested implants, to mimic the potential use in humans. After sacrifice, the implants (if not resorbed) were exposed and recovered. The subcutaneous injection of Bioregen^®^ did not lead to any visible plug, since the material was completely resorbed (n = 15 mice, three for each time point of 1, 2, 6 and 9 days). The addition of Bioregen^®^ to Matrigel (1:1, v:v) did not change the type of response with no evident angiogenesis induction (n = 6, data not shown).

**Figure 6:**
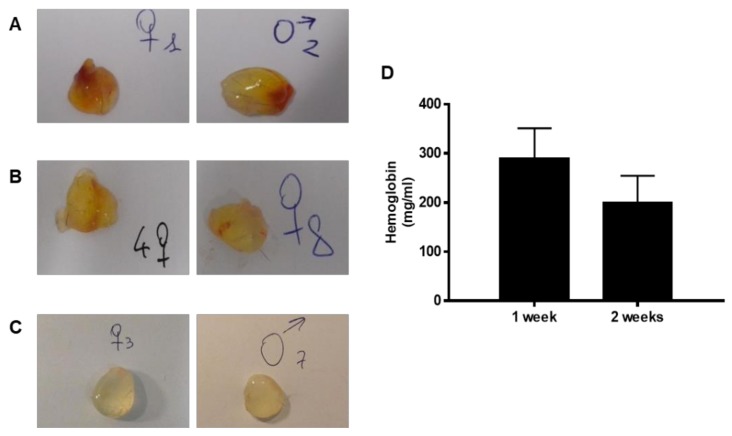
Representative images of the subcutaneous Bioexpander^®^ plugs recovered at different times. The pictures in A, B, and C report samples of the isolated subcutaneous implants. After one week (panels **A**), implants were clearly vascularized, while at two weeks vascularization was absent or partial, being present in some small spots (panels **B**). The content of haemoglobin (panel **D**) confirms reduction of vascularization. After four weeks (panels **C**), no implant was vascularized. For each time, two images are reported as representative of at least seven of the implants recovered.

Instead, the formation of a thin and transparent capsule was observed in all the implants of Bioexpander^®^ (implanted alone). In particular, after one week, in the seven plugs recovered out of eight implants performed, five implants were clearly vascularized (Figure 6A), one only partially and one not vascularized. After two weeks, seven plugs recovered out of the eight implants performed and the vascularization was absent or partially present in some small spots (Figure 6B). After four weeks, we recovered six implants out of eight (two implants were resorbed) and none of these implants were macroscopically vascularized (Figure 6C). The haemoglobin content confirmed these observations: at two weeks, the haemoglobin level decreased 50% with respect to one week, while at 4 weeks it was no longer detectable (Figure 6D). The same results on the induction of angiogenesis after one week were obtained when plugs were composed of 50% of Matrigel solution and 50% of Bioexpander^®^ (n = 5; data not shown).

**Figure 7:**
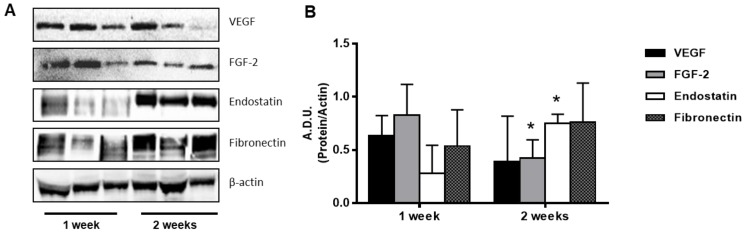
Western blot analysis of proteins in the subcutaneous plugs. Representative blots of samples at different times (panel **A**) and quantification of the signals of interest (panel **B**). Data are reported as A.D.U of the protein of interest with respect to β-actin. * *p* < 0.05 vs. 1 week samples.

The measurement of angiogenesis modulators in the plugs by Western blot indicated the presence of proangiogenic proteins (VEGF and FGF-2) after 1 week and their decrease at 2 weeks (Figure 7), with effects consistent with the haemoglobin content (Figure 6D). At the same time, the levels of the angiogenic inhibitor endostatin increased over time. The expression of endostatin, one of the most potent angiogenesis inhibitors, was tripled after two weeks (Figure 7) and remained high until 4 weeks (data not shown). In the complex, the balance of pro- and antiangiogenic modulators was in favor of «pro» in the first week and «anti» after a longer time. In the same plugs, the ECM protein fibronectin was already expressed in the first week, but variable among the samples.

In total, these data indicate the induction of an angiogenic response by Regenyal Idea Bioexpander^®^ when implanted subcutaneously, to an extent that was, however, modest and transient.

## 4. Discussion

In this paper, we describe the tolerability of two HA preparations with different chemico-physical features, and the proangiogenic behavior of Regenyal Idea Bioexpander^®^ when implanted subcutaneously, whereas the biomaterial is inert when implanted in the avascular tissue of the cornea. The neovascular response was evident in the first week before undergoing regression. VEGF and FGF-2 levels were high for the first week, while the global balance of the angiogenesis modulators moved toward inhibition after a longer time (after one week) with decreased VEGF and FGF-2 levels and increased expression of endostatin. Since in the avascular cornea tissue the biomaterial does not affect angiogenesis and the responses observed on cultured endothelial cells are modest and partial, it is suggested that reticulated HA containing low molecular weight molecules (namely Regenyal Idea Bioexpander^®^) in vivo induces the recruitment and activation of stromal cells, and is able to release factors that finely modulate the angiogenesis process.

It is widely reported in the literature that HA, due its structural role, that is, to keep tissues hydrated and maintain osmotic balance, regulates cellular processes such as adhesion, migration, and proliferation [1]. Different lengths of the HA polymers that are made up of repeated disaccharide units, D-glucuronic acid and N-acetyl-D-glucosamine, affect different cellular processes. The high molecular mass HA (≈106 Da) is anti-angiogenic, whereas HA fragments of 3–25 disaccharide units induce angiogenesis [27]. In this context, the Regenyal Idea Bioexpander^®^ does not induce proliferation of endothelial cells (HUVEC and HDBEC), but it is deposited on the cell surface in a concentration-dependent manner. Indeed, the biological functions of HA are mediated by cell surface HA receptors or HA-binding proteins [28]. HUVEC can bind HA in a dose-dependent manner; these sites are saturable and specific. The CD44 antigen, a type 1 transmembrane glycoprotein, is the main receptor for hyaluronan. Stimulation of CD44 triggers a signaling cascade that leads to increased cell growth, whereas c-Src kinase activity is responsible for the phosphorylation of cytoskeleton proteins and the induction of cell motility. In our hands, an increase in HUVEC migration at 0.1–1 mg/mL of low MW Bioexpander^®^ was demonstrated with no effect on cell proliferation. On the contrary, a slight increase in fibroblast proliferation was observed in skin fibroblasts, as previously demonstrated [29].

In this study, we observed that Regenyal Idea Bioexpander^®^ (and not Bioregen^®^) increases fibroblast and HUVEC migration. The most important receptor that triggers hyaluronan-mediated motility (RHAMM), also known as CD168, is present in several cell types including endothelial cells and various tumor cell lines [28]. Intracellular RHAMM interacts with cytoskeletal proteins, such as actin filaments and microtubules, and activates the previously mentioned protein kinases, which result in cell movement stimulation [30]. The reduction in the ECM protein fibronectin and the cell–cell contact marker ZO-1 corroborates the promigratory effect of Bioexpander^®^. These molecular features elicited by Bioexpander^®^ were shared also by Bioregen^®^, even if at functional level a promigratory effect is not evident. Probably, other mechanisms are involved in the final outcome of cell functional responses.

Literature data document that HA is a strong inducer of angiogenesis, although its biologic activity in tissues has been shown to depend on molecular size, chemical formula, and structure [27,31,32]. In the early stage of Regenyal Idea Bioexpander^®^ implantation, there is a reactive angiogenesis probably mediated through the recruitment of stromal cells that are able to locally increase the levels of proangiogenic factors. This is confirmed by the engagement of the CD40 receptor in endothelial cells that incites the production of adhesion molecules, cytokines, chemokines, and inflammatory mediators which are important for wound healing [33]. It has been widely reported in the literature that the binding of endothelial cell CD40 induces the expression of several proinflammatory cytokines as well as angiogenesis factors, including VEGF [12,34]. Our data on both fibroblasts and ECs confirm these findings, demonstrating both a direct proangiogenic effect on HUVEC (namely through an increase in the endogenous growth factor FGF-2) and indirect effect on fibroblasts (via the increase of VEGF) by Bioexpander^®^.

In summary, our study recounts the events of induction and activation of angiogenesis during a foreign body reaction (FBR). In general, the FBR resembles a sterile inflammatory reaction, i.e., inflammation in the absence of accumulating granulocytes, lymphocytes and plasma cells. Besides formation of a capsule and infiltration of cells such as macrophages into the biomaterial and fusion of these cells to giant cells, protrusion of neovascular sprouts into the biomaterial and surrounding tissue is a main characteristic of the FBR [35]. The macrophage population present immediately following tissue injury possesses predominantly M1 characteristics. Transition to an M2 phenotype occurs concurrently with resolution of the inflammatory process and the initiation of the remodeling phase of wound healing [16,36]. It is not excluded that these events occur in the subcutaneous implants over time. After one week, there is a spontaneous resolution of the inflammation state and for a long time the biodegradable, biocompatible, and bioresorbable properties prevail with concomitant angiogenesis reversion.

The mild inflammatory reaction could also participate in forming a layer of fibrous capsule on the scaffold, thus preventing its fast degradation. This explains why the Bioexpander^®^ implants are still visible after 1 month, while Bioregen^®^, which does not induce any change in the CD-40 in fibroblasts or endothelial cells, is completely resorbable in a short time.

## 5. Conclusions

From our in vitro models, we can confirm that the two HA preparations, one resorbable (Bioregen^®^) and the other reticulated and not immediately resorbable (Bioexpander^®^), have an optimal biocompatibility. Skin fibroblast functions and markers are maintained at a physiological level with HA preparations, with no significant modifications. Endothelial cells, being more susceptible to physical stimuli, show a positive response to the biomaterial only in a strict concentration range (0.1–1 mg/mL) with an activated angiogenic phenotype. When implanted subcutaneously, the reticulated biomaterial induces a transient proangiogenic behavior presumably linked to mild stromal/inflammatory cell activation.

Our data strengthen the beneficial characteristics of HA in aesthetic dermatology. These beneficial characteristics are mainly based on the properties of HA to bind a large number of water molecules, thus improving tissue hydration and resistance to mechanical stress [5,6].
